# The relationship between physical fitness and community participation in people with spinal cord injury

**DOI:** 10.4102/sajp.v73i1.354

**Published:** 2017-10-26

**Authors:** Linda van der Westhuizen, Diphale J. Mothabeng, Tshifhiwa M. Nkwenika

**Affiliations:** 1 Department of Physiotherapy, Faculty of Health Sciences, University of Pretoria, South Africa; 2 South African Medical Research Council, South Africa

## Abstract

**Background:**

People with spinal cord injury (PWSCI) who use wheelchairs for mobility tend to be physically inactive because of their limited mobility. Poor endurance and exercise tolerance, associated with poor physical fitness, can make it challenging to meet the physical demands of activities such as manoeuvring a wheelchair over gravel roads. This may lead to poor community participation in activities PWSCI were involved in pre-morbidly. To date, no studies have been conducted in South Africa on what the relationship is between physical fitness and community participation in PWSCI.

**Aim:**

The purpose of this study was to establish the relationship between physical fitness and community participation in PWSCI.

**Methodology:**

An exploratory cross-sectional survey was conducted on PWSCI living in the Greater Tshwane Metropolitan City. Physical fitness was measured using the 6 minute push test (6MPT) and the Borg scale. Community participation was measured using the Reintegration to Normal Living Index (RNLI). The data were analysed using the Spearman’s Rank correlation at a 5% level of significance.

**Results:**

Moderate to poor associations were found between the 6MPT and the Borg scale with the RNLI (*r* = 0.637; *p* < 0.001 and *r* = −0.325; *p* = 0.013, respectively). These results indicate that the participants who were able to push further in 6 min and had better endurance were more satisfied with their perceived community participation.

**Conclusion:**

This study shows that there is a relationship between physical fitness and community participation in PWSCI. Information gained from this study lays the foundation for more studies in this area, and for possible improvement in rehabilitation practice.

## Introduction

Spinal cord injury (SCI) is a traumatic and life changing event, which leads to neurological deficits and disabilities, because of the loss of mobility and sensation (Lee et al. 2011; Silva et al. 2014). The loss of mobility associated with SCI is a major contributing factor influencing inactivity in people with spinal cord injury (PWSCI) (Wendell et al. 1998). It has been found that inactivity is associated with SCI, and PWSCI are more likely to lead physically inactive lives than most other populations (Gorgey 2014; Letts et al. 2011; Mothabeng et al. 2008; Njoki, Frantz & Mpofu 2007). A close relationship exists between physical fitness and physical activity, where living an active lifestyle promotes good physical fitness (Gielen, Schuler & Volker 2010). Living a physically active life promotes physical fitness and has been found to improve the quality of life (QoL), both objectively and subjectively in PWSCI (Tomasone et al. 2013; Warburton et al. 2007).

The participation of PWSCI in areas such as work and community activities has a direct influence on their perceived QoL (Ulrich et al. 2012). The more PWSCI are involved in these types of activities, the better they perceive their QoL to be. People with SCI often find it challenging to return to the pre-morbid roles they fulfilled in their community (Gorzkowski et al. 2011; Kuipers et al. 2011), and this may partially be because of new challenges of mobility after an SCI (Gorzkowski et al. 2011). Fatigue during mobility is encountered because of a lack of endurance needed to keep up with physically demanding environments (e.g. uneven outdoor terrain) encountered when participating in community activities (Gorzkowski et al. 2011). Poor physical fitness has also been found to be associated with poor upper limb strength, which is essential in people using wheelchairs for mobility, as they are reliant on their arms (Widman et al. 2007). Poor upper limb muscle strength may also contribute to higher levels of fatigue during activities, making mobility and community participation difficult. Poor endurance may result in withdrawal from society, decreased community participation and decreased QoL.

Individuals who are reliant on using wheelchairs for mobility are at a higher risk of physical inactivity and poor physical fitness because of limited mobility (Mothabeng et al. 2008; Wendell et al. 1998). Physical fitness can play a prominent role in minimising fatigue and has a direct relationship with independent wheelchair mobility (Hetz, Latimer & Martin Ginis 2009).

This study therefore aimed to investigate the relationship between physical fitness, which is associated with physical activity, and community participation in PWSCI who live in the Greater Tshwane Metropolitan (GTM) City, Gauteng, South Africa.

## Method

### Participants

Potential participants were identified from the database of a private rehabilitation centre located in the GTM, Gauteng, South Africa. Sixty volunteers constituted the sample of convenience after they met the following requirements:

- were between 18 and 70 years of age
- had a medical diagnosis of SCI
- used a manual wheelchair for their primary means of mobility
- were able to push their wheelchair independently
- had been living in the community for at least 6 months post-discharge from the spinal rehabilitation setting.

The sample size was calculated using 10 participants for every one of the six objective outcome measures used in the study on which this paper is based. This is in accordance with sample size requirements by Connelly (2008).

### Procedure

All measurements were conducted and recorded by the first author, except in the instances where a language barrier existed and an interpreter was required. The interpreter was trained as to how to apply all the data collection tools prior to data collection to prevent any errors in translation.

Demographic data were gathered using the Socio-Demographic and Injury Profile (SDIP). The components of the SDIP used in this paper included age, gender and details about the SCI. This was verbally administered to the participants.

The Reintegration to Normal Living Index (RNLI) was used to determine the perceived satisfaction of community participation (Spinal Cord Injury Rehab Evidence (SCIRE) 2013). The RNLI was verbally administered to the participants and consists of 11 statements, which address seven different areas: indoor, community and distance mobility; self-care; daily activities of work and school; recreational and social activities; family role(s); personal relationships; and presentation of self to others. Each individual statement on the RNLI is scored from 0 to 10, with a score of 0 not being at all like their life situation and 10 being exactly what they are currently experiencing in day-to-day life. The total score of the RNLI is then converted into a percentage in order to categorise the participants’ perceived community participation. These categories include full participation (100%); mild to moderate restrictions (60%–99%); and severe restrictions (less than 60%), with perceived community participation (Pang, Eng & Miller 2007). Stark et al. (2005) found the RNLI very reliable to use in individuals living with permanent physical effects from chronic conditions with an intraclass correlation coefficient of 0.91. Mothabeng, Eksteen and Westaway (2012) found the RNLI reliable to use in PWSCI living in South Africa (Cronbach’s alpha for the RNLI instrument 0.97 and an intraclass correlation coefficient of 95%).

The 6 minute push test (6MPT) was used to determine physical fitness. The 6MPT is derived from the 6 minute walk test (Cowan, Callahan & Nash 2012). It involves the individual pushing a manual wheelchair, over a flat surface, as far and hard as they can in 6 min (Enright 2003). Distances of 604 m for people who are paraplegic and 445 m for people who are tetraplegic indicate good fitness levels (Cowan et al. 2012). The 6MPT has been shown to be highly reliable and sensitive to determine cardiovascular fitness in individuals with SCI, with an intraclass correlation coefficient of 0.9 (*p* < 0.05) (Cowan et al. 2012).

A 50 m distance, at or nearby the participants’ homes, was measured with a measuring tape over a flat surface, for the 6MPT. The location of this measured distance was the nearest paved surface or local community hall to the participant. The participant was then instructed to push as many of the measured 50 m lengths as possible in 6 min, which was timed with an electronic stopwatch. The participant was permitted to rest when needed, but was encouraged to push as much and fast as he or she could. The stopwatch was not paused during any rest breaks taken by the participant. The total distance the participant completed was then calculated. This distance was noted, and recorded.

Directly after the 6MPT, the participant was asked to complete the Borg scale (also known as the Rating of Perceived Exertion Scale), which was developed by Borg (1998), to subjectively measure the extent of exertion after increased physical activity (6MPT). This was also recorded. The Borg scale uses a visual analogue scale to indicate the level of exertion experienced, zero being ‘nothing at all’ and 10 being ‘very, very hard’ (Maximal) (Borg 1998). The Borg scale has been shown to have high reliability (intra-rater reliability = 0.94) and validity (*r* = 0.80–0.90) (Chen, Fan & Moe 2002). Chen et al. (2002) found that the Borg correlates well with respiratory rate (0.72) and heart rate (0.62) in levels of exertion in able-bodied participants.

All data collected were captured onto an Excel spreadsheet in preparation for analysis.

### Data analysis

Data were analysed using Stata14. The *t*-test was used to determine the relationship of gender and type of lesion with the 6MPT, the Borg scale and the RNLI. The correlation between the physical activity variables (6MPT and the Borg scale) and the community participation outcome measure (RNLI) was calculated using the Spearman’s Rank coefficient.

### Ethical considerations

An exploratory cross-sectional survey was conducted on PWSCI living in the GTM. The study was granted ethical clearance by the University of Pretoria Health Sciences Research Ethics Committee (Ethics reference number: 192/2014).

## Results

Sixty PWSCI participated in the study. Their socio-demographic profiles are described according to gender, age and type of SCI in Table 1. The majority of the participants were male (83.33%), had a mean age of 38.4 years and had paraplegic-type injuries (65%) with complete SCI [American Spinal Injury Association (ASIA) Impairment Scale] (81.66%).

**TABLE 1:** Socio-demographic profile of participants.

Socio-demographic characteristics	*n* (%)
Gender	Female = 10 (16.67%) Male = 50 (83.33%)
Age at the time of the study	Ages ranged from 18 to 67 years Mean age = 38.4 years
ASIA classification	ASIA A (complete injury) = 49 (81.66%) ASIA B (incomplete injury) = 6 (10%) ASIA C (incomplete injury) = 4 (6.66%) ASIA D (incomplete injury) = 1 (1.66%)
Type of spinal cord injury	Paraplegics = 39 (65%) Tetraplegics = 21 (35%)

*Source*: Authors’ own work

An independent samples *t*-test was conducted to compare RNLI scores of male and female participants. As illustrated in Table 2, there was a significant difference in the scores of the male (*M* = 96.3, SD = 14.3) and female (*M* = 84.4, SD = 25.7) participants (*p* = 0.041). These results suggest that gender has an effect on satisfaction with participation.

**TABLE 2:** Differences between male and female participants on the reintegration to normal living index.

Group	No. of participants	Mean (±) RNLI score	95% conf. interval	
Male	50	96.3 (14.31)*	92.23	100.37
Female	10	84.4 (25.18)*	66.39	102.41

*Source*: Authors’ own work

RNLI, reintegration to normal living index.

* , *p* = 0.04.

The mean distances pushed on the 6MPT are illustrated according to the type of injury in Table 3 and gender in Table 4. The participants with paraplegia pushed further distances compared to those with tetraplegia. The male participants were also able to push further on average compared to the female participants.

**TABLE 3:** The 6 minute push test distances pushed according to type of injury.

Type of injury	Mean distance	Min	Max	Expected mean distance according to Cowan et al. (2012)
Paraplegia	692.92 m	252 m	1412 m	604 m
Tetraplegia	380.86 m	30 m	868 m	445 m

*Source*: Authors’ own work

**TABLE 4:** The 6 minute push test distances pushed according to gender.

Gender	Mean distance	Min	Max
Male	608.08 m	30 m	1412 m
Female	430.7 m	200 m	744 m

*Source*: Authors’ own work

The participants’ perceived exertion according to the Borg scale is illustrated in Table 5 according to the type of injury. The participants with paraplegia were less exhausted after the 6MPT with a mean score of 3.5 on the Borg scale, while those with tetraplegia had a mean score of 5.5. The highest individual score, however, was also reported by a participant with tetraplegia.

**TABLE 5:** The Borg scale according to type of injury.

Type of injury	Mean Borg scale	Min	Max
Paraplegia	3.5	1	7
Tetraplegia	5.5	2	10

*Source*: Authors’ own work

A moderately positive statistically significant direct relationship between the 6MPT and the RNLI (*r* = 0.637; *p* < 0.001) was found (Table 6). These results indicate that participants who were able to push further distances in the 6MPT were more satisfied with their perceived community participation, as described by the RNLI.

**TABLE 6:** Relationship between reintegration to normal living index, the 6 minute push test and Borg scales.

Relationship	*r*	*p*
RNLI and 6MPT distance	0.637	< 0.001
RNLI and Borg scale	−0.325	0.013

*Source*: Authors’ own work

RNLI, reintegration to normal living index; 6MPT, 6 minute push test.

On the other hand, there was a weak inverse statistically significant relationship between the Borg scale and the RNLI (*r* = −0.325; *p* = 0.013) (Table 6), indicating that participants who experienced less exertion after the 6MPT were more satisfied with their perceived community participation, as described by the RNLI.

## Discussion

The aim of this study was to identify whether there exists a relationship between physical fitness and community participation in PWSCI.

The majority of the participants in this study were male. Similar trends have been reported locally by Hart and Williams (1994) and Mothabeng (2011). The mean age of the participants at the time of the study was 38.4 years, ranging from 18 to 67 years of age. The majority of the participants (80%) had an ASIA A complete injury, with the remaining participants falling into the incomplete classifications (B, C and D), similar to other local studies (Hart & Williams 1994; Mothabeng 2011). Of the participants, 65% presented with paraplegia and 35% with tetraplegia. This is in accordance with studies that show there is a global trend of about 60% of SCI being paraplegic and 40% quadriplegic (Hetz et al. 2009; Rahimi-Movaghar et al. 2013; Wyndaele & Wyndaele 2006).

The RNLI results suggest that, on average, participants in this study were satisfied with their community participation as they experienced mild to moderate restrictions to community participation, with a mean score of 94.3/110 (85.73%). Participants with paraplegia were much more satisfied with their community participation compared to the participants with tetraplegia. This may be because of people with tetraplegia experiencing higher levels of disability, making mobility in the community much more difficult than it is for paraplegics (Urbanski, Bauerfeind & Pokaczajlo 2013).

Both male and female participants fell into the mild to moderate restrictions category (60%–99%), although the male participants experienced fewer restrictions to community participation. The male participants had a total RNLI score of 87.54% (96.2/110) and the female participants had a total RNLI score of 76.27% (84.4/110). Men with SCI tend to be more actively involved in the community compared to their female peers (Rauch et al. 2013). This may be because of the fact that in PWSCI, who are reliant on wheelchairs for mobility, men tend to be physically stronger and mobilise around the community easier than women (Frontera et al. 1991). Women also tend to have more home-based responsibilities that leave them with less time for community participation, which may also be an influencing factor for their poorer satisfaction with their community participation (Rauch et al. 2013).

It is therefore not surprising that the majority of male participants had good physical fitness levels (mean distance = 608 m) and the majority of female participants had low physical fitness levels (mean distance = 430 m). The male participants were able to push a considerably greater distance in the 6MPT than the female participants, with a mean difference of 177 m. The maximum distance pushed by the male participants was also almost twice the maximum distance pushed by the female participants. This may be because of the fact that men are physiologically stronger than women (Frontera et al. 1991) and, therefore, propel their wheelchairs with more ease than their female counterparts. Another study found that in PWSCI females tend to be more inactive than their male counterparts (Rauch et al. 2013).

Average distances pushed during the 6MPT by participants with paraplegia and tetraplegia were 693 m and 381 m, respectively. Cowan et al. (2012) determined that 604 m for paraplegics and 445 m for tetraplegics indicated good physical fitness levels, suggesting that participants with paraplegia had good physical fitness levels and participants with tetraplegia had low physical fitness levels. The majority of the participants (56.6%) in this study had a low physical fitness level, with 16 of the 22 participants with tetraplegia pushing under 445 m, and 18 of the 38 participants with paraplegia pushing under 605 m. This is similar to studies that found that PWSCI with higher levels of SCI (tetraplegics) have greater challenges with mobility when compared to those with lower levels of SCI (paraplegics) (Hetz et al. 2009; Urbanski et al. 2013).

The participants who were able to push further distances in the 6MPT showed a higher satisfaction with community participation according to the RNLI. The moderate relationship between the 6MPT and the RNLI may be because of the fact that the participants who were able to push further distances had better upper limb strength (i.e. those with paraplegia) and more endurance compared to the participants who pushed shorter distances. This, in turn, may lead to overcoming obstacles, such as car transfers, uneven terrains and curbs encountered during community mobility with a wheelchair. This made it more likely for these participants to be involved in community activities (Gorzkowski et al. 2011), thus improving their QoL (Ulrich et al. 2012). This finding is similar to that of studies conducted by Gorgey (2014), Mackie, McCormack and Campbell (1989), Rauch et al. (2013), Thompson (2003) and Widman et al. (2007) who found that endurance and good upper limb strength are essential for PWSCI to improve mobility and QoL.

The majority of the participants (57%) reported high to maximal levels of exhaustion on the Borg scale after the 6MPT. Studies have shown PWSCI tend to be more physically inactive and have poorer cardiovascular fitness levels than other populations (Van der Woude et al. 2013).

There was a poor negative association between the level of exertion (according to the Borg scale) after the 6MPT and satisfaction with community participation. The participants who reported lower levels of exertion after the 6MPT tended to be more satisfied with their community participation. This increased satisfaction with community participation may be because of the fact that participants who experienced lower levels of exertion have better endurance, which makes mobilising in the community easier compared to participants with lower endurance, leading to higher levels of community participation (Gorzkowski et al. 2011). This adds to the above statement on distances pushed during the 6MPT, that good endurance is a major factor in improving community mobility and independence in PWSCI (Mackie et al. 1989; Widman et al. 2007).

The study findings have highlighted the importance of physical fitness in participation. Therefore, rehabilitation programmes need to include fitness training and ensure that patients are ready for community living before discharge. It is important to raise awareness about the positive influence of physical fitness and endurance on community participation and QoL in PWSCI. Education on the importance of physical fitness and assisting PWSCI to implement strategies to be more physical active, from the initial physical rehabilitation phase as well as in the community, may be valuable.

### Limitations

The sample size of this study was limited because of the small population of PWSCI who live in Tshwane and were available to participate in the study. This can potentially have an influence on the results of the outcomes when compared to the general population of PWSCI.

Only participants from the private sector were involved in this study. This may influence the study in that many of the participants making use of private rehabilitation come from more financially advantaged backgrounds and have greater access to resources to assist with physical activity.

## Conclusion

This exploratory cross-sectional survey found that PWSCI who have better physical fitness experience higher satisfaction with their perceived community participation, and ultimately better community participation than those who have poor physical fitness and endurance. Further studies as to how physical activity (including the implementation thereof) can improve community participation in PWSCI are recommended.
